# Inflammatory Septal Nasal Polyp

**Published:** 2015-07

**Authors:** Neha Salaria, Naveen Sharma, Uma Garg, Swaran Kaur Saluja, Ruchi Agarwal

**Affiliations:** 1 *Department of Otorhinolaryngology, BPS Government Medical College for Women, Khanpur Kalan, Haryana, India. *; 2 *Department of Pathology, BPS Government Medical College for Women, Khanpur Kalan, Haryana, India.*

**Keywords:** Inflammatory, Septal, Polyp.

## Abstract

**Introduction:**

Nasal polyps are benign prolapsed mucosal lesions which commonly arise from the paranasal sinuses and lateral walls of the nose especially the contact areas of osteomeatal complex. Though nasal polyps arising from the nasal septum have been reported, those arising from its anterior part are extremely rare and present a diagnostic dilemma. Aetiology is multifactorial and is mainly a result of the inflammatory response of the lining mucosa.

**Case Report:**

The case of a 28-year-old male, with a history of progressively increasing nasal complaints since 4 months and with a nasal mass arising from the anterior nasal septum on examination, is reported. Diagnosis of an inflammatory nasal polyp was made on histopathological examination after surgical excision of the mass.

**Conclusion:**

The diagnosis of an inflammatory nasal polyp was not only unusual in terms of its location but also in its appearance on anterior rhinoscopy and tomographic scanning images. The definitive diagnosis in such cases can only be achieved through surgical resection and detailed histopathological examination.

## Introduction

Nasal polyps are benign abnormal mucosal protrusions associated with nasal mucosa or paranasal sinuses. They were first reported 4000 years ago. Hippocrates referred to these nasal masses as “polypus” due to its resemblance to the sea–polyp (many footed). They appear as fluid filled semitransparent tear drop-like structures. The prevalence in the adult population is estimated to be around 1-4% (1).

Increasing prevalence suggests multiple factors contributing to its pathogenesis. Nasal polyps are thought to result from inflammation of the mucosa resulting from allergy or even from anatomic obstruction because of altered air currents and increased air pressure. Polyps may be a presenting feature of fungal sinusitis, allergic rhinitis, asthma, ciliary dyskinesias, or aspirin intolerance. 

Polyps may arise from the mucosa of the nose or paranasal sinuses. The most commonly involved sinuses are ethmoids, maxillary, sphenoid, and frontal, respectively in that order. These may also arise in the middle meatus from the uncinate process and the ethmoidal infundibulum. Ethmoid and antral polyps need important mention. Ethmoidal polyps are allergic in nature and their clinical spectrum may extend from a limited disease, which responds to conservative treatment, to a very extensive one, which shows frog faced deformity and requires functional endoscopic sinus surgery or even multiple surgeries. Unilateral polyps are most commonly described as antrochoanal polyps, but there are other disorders which need to be evaluated: these are mainly Inferior turbinate hypertrophy, inverted papilloma, septal papilloma, big mucosal blob, olfactory neuroblastoma, rhinosporidiosis, rhinoscleroma, juvenile nasal angiofibroma, or angiofibroma of the septum. However, solitary lesion arising from the anterior nasal septum, as in the case presented here, is extremely rare and can present a diagnostic dilemma requiring radiological and histological assistance to conclude the diagnosis.

## Case Report

A 28-year-old male with a history of progressively increasing nasal obstruction since 4 months, was presented to the clinic. This nasal obstruction was associated with a mass in the nasal cavity seen through the anterior nares, which was initially small and probably arose from the septum, but had gradually increased to obstruct almost the whole right nasal cavity. There was no history of nasal discharge, post nasal drip, allergy, epistaxis, headache, facial fullness, alteration in smell, or other nasal complaints. There was broadening of the right nasal cavity with prominence of the right nasal vestibule. 

**Fig 1:**
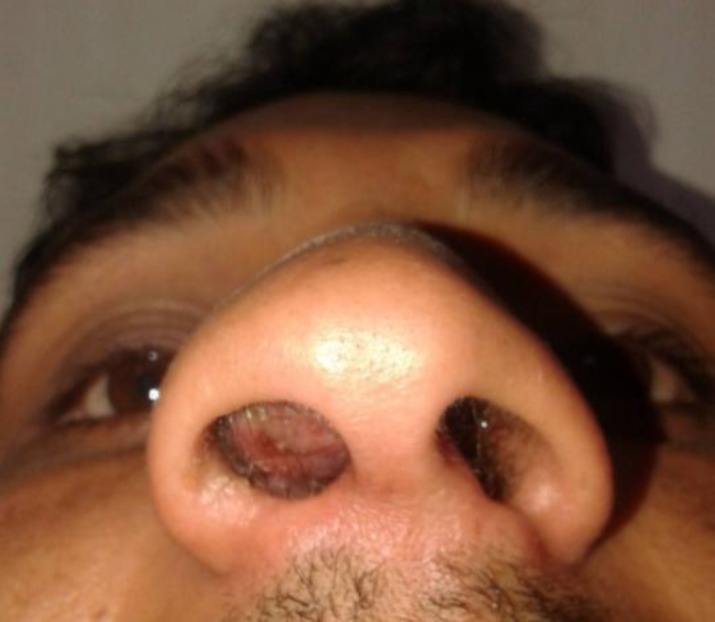
Preoperative basal view (note the assymetry of the nares

On anterior rhinoscopy a pinkish mass was seen filling the right nasal cavity (Fig. 1). 

When probing the mass; which was attached to the septum, vestibule, and roof with a broad pedicle; it was observed to be firm in consistency, non friable, non tender, and did not bleed on touch. Posterior rhinoscopy was insignificant. Detailed nasal endoscopy revealed that the middle meatus was clear and there was no other mass or anomaly. The left nasal cavity was within normal limits. The rest of the otolaryngological examination did not reveal any noteworthy abnormality. A probable diagnosis of a granulomatous mass, septal papilloma, hemangioma, polyp, and rhinoscleroma was made.

Fine needle aspiration cytology of the mass suggested abundant eosinophilic cells and a differential diagnosis of nasal polyp, eosinophilic granuloma, and eosinophilic angiocentric fibrosis was considered. 

**Fig 2:**
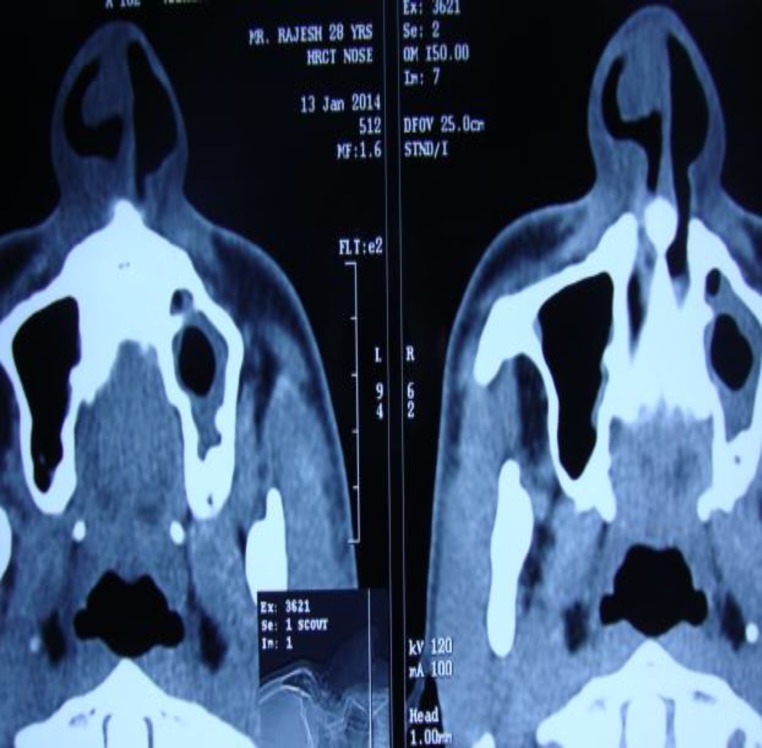
CT Scan (Axial section) shows a mass arising from the septum

CT Scan confirmed the presence of a right nasal mass arising from the anterior part of the nasal septum and the adjoining roof, which was obstructing the right nasal cavity. Aeration of the associated sinuses were clear (Fig. 2). 

**Fig 3:**
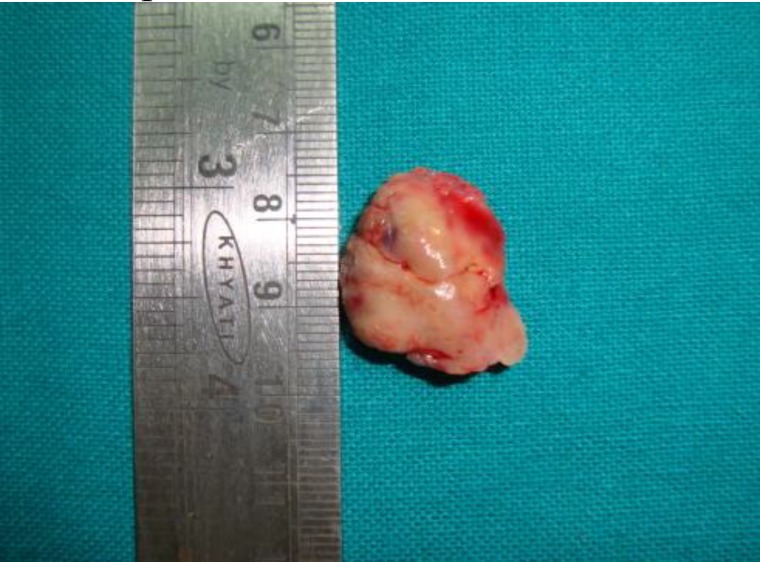
Gross specimen was excised

Prior to surgical intervention an informed consent was taken. The patient was put under local anaesthesia using 1:100000 lignocaine in adrenaline and, using endoscopic guidance, the mass was excised with an adjacent margin of normal healthy tissue. The specimen macroscopically measured 2.5 X 2 cm in size, was pink in colour, firm in consistency, and had a lobulated smooth surface (Fig.3). 

**Fig 4:**
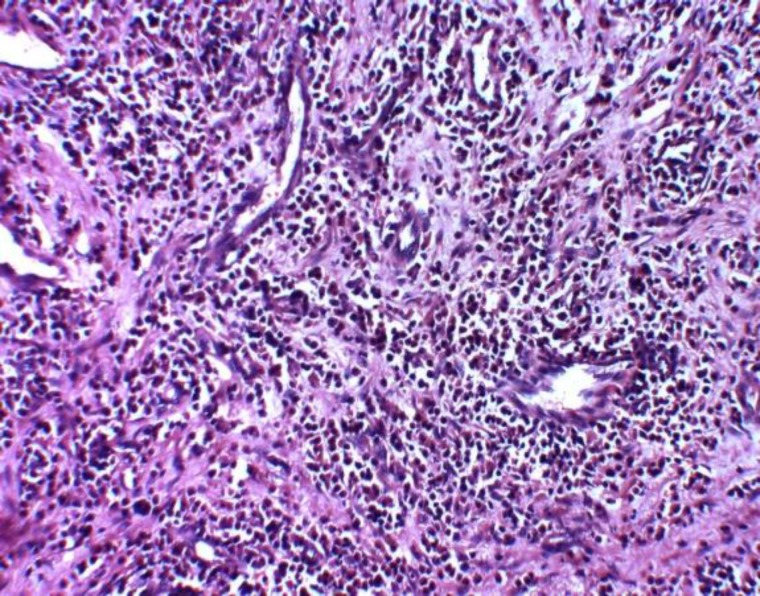
Microphotograph shows polymorphic inflammatory infiltrate with predominance of eosinophils (H&E X 200)

The mass was sent for histopathological examination, which suggested features consistent with an inflammatory nasal polyp (Fig.4). The postoperative period was uneventful and the patient is on regular follow up with no recurrence.

## Discussion

Traditionally nasal polyps are said to arise from the mucosa of the sinus and enter the nasal cavities through the ostium. They are mainly classified into antral and ethmoidal types depending on the sinus of origin. Nasal polyps may also arise from the middle turbinate, ethmoidal infundibulum, and contact areas of the uncinate process. However, a review of literature suggests polyps from various other unusual sites i.e posterior nasal septum, floor of the nose, cribriform plate, and sphenoid sinus have been reported (2-6). 

The first case of septal polyp was reported by Bailey in 1979 (8). Stammberger, in a study on 200 patients, reported only 3 cases of polyps arising from the posterior nasal septum (9). De Stefano et al reported 2 cases of septal polyps in 150 cases of FESS: one of them was associated with allergy, while the other was associated with a deviated septum (6). 

The exact aetiopathogenesis is not known and several theories have been postulated in the past 150 years. Allergy and chronic inflammation are commonly implicated factors. Some of the proposed mechanisms include the adenoma and fibroma theory by Billroth, an increase in tubuloalveolar glands, structural defects like deviation of the nasal septum, mucosal oedema, mucosal inflammation , and epithelial rupture. More recently the role of inflammatory mediators like Interleukins (3,5,8), transforming growth factor beta, basic fibroblast growth factor, leptin, and HPV-11 have been evaluated (10).

Bernstein proposed the multivariate theory for the pathogenesis of polyps (11). Preponderance of polyps are seen in the contact area of the lateral wall of the nose especially in patients with obstruction of the middle meatus, which acts as a drainage site of sinuses. This is due to increased air turbulence in this area, compared to the lower third of the nose, and viral-bacterial-host interactions trigerring ulceration and subsequent reepithelialisation and neo gland formation. As a result of the heightened release of cytokines and inflammatory mediators, the integrity of the sodium potassium channel is disrupted causing sodium absorption, water retention, and polyp formation (11). Gravity and negative pressure are also thought to play a role in enlarging polyps (10). 

In rare cases, like the one presented here, where the site of origin is from an unusual site like the anterior nasal septum, the exact cause is still unknown. Vinayak S reported a similar case of an allergic polyp of the ala of the nose associated with a deviation of the nasal septum (12). In the current case, there was no deviation of the nasal septum or any other obstruction and no history suggestive of allergy or any ciliary dyskinesias. However, this could probably be due to chronic irritation such as trauma due to a finger nail acting as a nidus, which stimulates chronic inflammation and causes polyp formation.

Diagnosis of nasal polyps is by anterior rhinoscopy and a detailed endoscopic examination is necessary especially in cases where the site of origin is rare. Preoperative nasal endoscopy is essential to make a tentative diagnosis for further investigations and for planning the surgical course. A preoperative CT scan is essential to confirm the diagnosis, to evaluate the extent of the pathology, and to rule out anatomical variations contributing to the origin. 

Treatment of nasal polyps include an adequate trial of corticosteroids that can be taken orally as well as in the form of nasal sprays. In the case of resistant polyps, endoscopic sinus surgery is the treatment of choice. In cases of isolated septal polyp, excision with a wide healthy margin gives good results. The postoperative outcome was favourable in this case: there was no recurrence and the patient is on regular follow up.

## Conclusion

To conclude, the diagnosis of an inflammatory nasal polyp was not only unusual in terms of its location but also in its appearance observed in anterior rhinoscopy and tomographic scanning images. The definitive diagnosis in such cases can only be achieved through surgical resection and detailed histopathological examination. Further research should be directed to evaluate definitive pathogenesis of polyps; including the role of chemical mediators, triggering factors, and genetic susceptibility; as it may play an important role in evolving future management strategies.
